# O1-conotoxin Tx6.7 cloned from the genomic DNA of *Conus textile* that inhibits calcium currents

**DOI:** 10.1590/1678-9199-JVATITD-2022-0085

**Published:** 2023-05-22

**Authors:** Maojun Zhou, Manyi Yang, Huiling Wen, Shun Xu, Cuifang Han, Yun Wu

**Affiliations:** 1 Department of Oncology, NHC Key Laboratory of Cancer Proteomics, State Local Joint Engineering Laboratory for Anticancer Drugs, Xiangya Hospital, Central South University, Changsha, Hunan, China.; 2 Department of Hepatobiliary and Pancreatic Surgery, NHC Key Laboratory of Nanobiological Technology, Xiangya Hospital, Central South University, Changsha, Hunan, China.; 3 School of Pharmacy, Gannan Medical University, Ganzhou, Jiangxi, China.; 4 Guangdong Provincial Key Laboratory of Medical Molecular Diagnostics, Guangdong Medical University, Dongguan, China.

**Keywords:** conotoxin, Tx6.7, calcium currents, hCaV1.2, hCaV2.2, Conus textile

## Abstract

**Background:**

Conotoxins exhibit great potential as neuropharmacology tools and therapeutic candidates due to their high affinity and specificity for ion channels, neurotransmitter receptors or transporters. The traditional methods to discover new conotoxins are peptide purification from the crude venom or gene amplification from the venom duct.

**Methods:**

In this study, a novel O1 superfamily conotoxin Tx6.7 was directly cloned from the genomic DNA of *Conus textile* using primers corresponding to the conserved intronic sequence and 3’ UTR elements. The mature peptide of Tx6.7 (DCHERWDWCPASLLGVIYCCEGLICFIAFCI) was synthesized by solid-phase chemical synthesis and confirmed by mass spectrometry.

**Results:**

Patch clamp experiments on rat DRG neurons showed that Tx6.7 inhibited peak calcium currents by 59.29 ± 2.34% and peak potassium currents by 22.33 ± 7.81%. In addition, patch clamp on the ion channel subtypes showed that 10 μM Tx6.7 inhibited 56.61 ± 3.20% of the hCa_V_1.2 currents, 24.67 ± 0.91% of the hCa_V_2.2 currents and 7.30 ± 3.38% of the hNa_V_1.8 currents. Tx6.7 had no significant toxicity to ND7/23 cells and increased the pain threshold from 0.5 to 4 hours in the mouse hot plate assay.

**Conclusion:**

Our results suggested that direct cloning of conotoxin sequences from the genomic DNA of cone snails would be an alternative approach to obtaining novel conotoxins. Tx6.7 could be used as a probe tool for ion channel research or a therapeutic candidate for novel drug development.

## Background

Conotoxins are small marine peptides typically comprising 10-50 amino acids and 1-5 disulfide bridges, which are produced by marine mollusks known as cone snails [ 1]. There are over 700 species in the genera of *Conus* [ 2, 3]. Each *Conus* species may contain up to 1000 conotoxins, which makes the library of bioactive conotoxin peptides very large [ 4, 5], perhaps over 140,000 different peptides in total. Conotoxins act potently and selectively on a lot of membrane receptors and ion channels, thus showing great potential as neuropharmacology tools and therapeutic candidates, and one calcium channel inhibitor, ω-conotoxin MVIIA (Prialt^TM^), was developed as a drug for treating neuropathic pain [ 6- 8]. 

According to the similarities of signal region sequences, conotoxins can be divided into 30 gene superfamilies [ 9]. The O1 superfamily which was first named in 1995, mainly contains the cysteine framework VI/VII (-C-C-CC-C-C-) and can be divided into the κ, δ, ω and μO pharmacological families [ 10]. At present, the δ and ω families have been found only in the O1 gene superfamily, which is the most abundant superfamily and contains more than 700 conotoxin sequences [ 11]. κ-PVIIA targeting voltage-gated potassium channels is the only O1-conotoxin belonging to the κ family [ 12]. O1-conotoxins targeting voltage-gated sodium channels belong to the δ and μO families [ 13]. The μO family conotoxins are sodium channel antagonists, while the δ family conotoxins are sodium channel agonists that delay the inactivation of sodium channels. The ω family conotoxins, such as ω-conotoxin MVIIA (Prialt^TM^), are blockers of voltage-gated calcium channels [ 14], suggesting that obtaining novel O1 superfamily conotoxins is important for the development of new analgesic drugs.

In a previous study, we presented the gene structures of nine conotoxin superfamilies, which have similar architectures with two introns and three exons [ 15]. For conotoxins in the same superfamily, the beginning and ending portions of the intronic sequences are conserved [ 16, 17], so it becomes possible to clone novel conotoxin sequences from the genome using primers corresponding to the 3’ UTR and 3’ end of the intron. In this study, we report a novel O1-conotoxin Tx6.7, which was directly cloned from the genomic DNA of *Conus textile*. The effects of Tx6.7 were tested on sodium, potassium and calcium currents in DRG neurons and ion channel subtypes (hNa_V_1.3, hNa_V_1.4, hNa_V_1.7, hNa_V_1.8, hCa_V_1.2 and hCa_V_2.2) in HEK293 cells or ND7/23 cells. Tx6.7 was also tested for cytotoxic activities on ND7/23 cells and analgesic activities by the mouse hot plate assay. 

## Methods

### Specimen collection and genomic DNA cloning

Specimens of *Conus textile* were collected from reef flats on West Island near Sanya, China. Genomic DNA of *Conus textile* was prepared from 30 mg of frozen tissue (muscular foot) using the E.Z.N.A.^TM^ Mollusc DNA Kit (OMEGA, America) according to the manufacturer’s standard protocol. Genomic DNA PCR was performed to obtain the gene sequences of O1-conotoxins. The primers were designed based on the 3’ end of the intron preceding the mature peptide region and the 3’ UTR elements conserved in the O1 superfamily (forward primer: 5'-CGATCCATCTGTCCATCCATC-3'; reverse primer: 5'-GAKGGGAGTAGAACACATCACTA-3'). The amplified PCR products were extracted and cloned. DNA sequencing was carried out using an ABI 3730 automated DNA Analyzer (Thermo Fisher Scientific, America). 

### Peptide synthesis of Tx6.7

The mature peptide of conotoxin Tx6.7 was synthesized on a Rink amide resin using a standard Fmoc strategy according to previously reported methods [ 17]. The three pairs of cysteines were protected by triphenylmethyl (Trt), acetamidomethyl (Acm), or methoxybenzyl (Mob) separately and the three disulfide bonds were successively formed by oxidation of oxygen, iodine, or potassium ferricyanide. After oxidation, the mature peptide was purified by reverse-phase high-performance liquid chromatography (RP-HPLC), and the molecular weight was confirmed by mass spectrometry analysis. After purification by HPLC, the purity of synthetic Tx6.7 was more than 98%.

### Whole-cell patch clamp for DRG cells

Acutely separated DRG cells were isolated as previously described [ 18]. SD rats (30 days old) were purchased from the Guangzhou University of Chinese Medicine Experimental Animal Center (NO. SYXK (Yue) 2018-0182). All animal procedures were carried out according to the approved protocol (GDY2002208) of the Institutional Animal Care and Use Committee at Guangdong Medical University. The rats were euthanized, and the dorsal root ganglia tissue was removed quickly and cut into small pieces. The ganglia were treated with 0.1% collagenase and 0.05% trypsin. After centrifugation, the DRG cells were suspended in essential DMEM with 10% (v/v) fetal bovine serum and incubated in a 5% CO_2_ fully humidified environment at 37 °C.

For recording sodium currents, the intracellular solution contained the following composition: 10 mM CsCl, 5 mM NaCl_2_, 10 mM HEPES, 2 mM Mg-ATP, 135 mM CsF, and 5 mM EGTA, pH = 7.2 (CsOH), and the extracellular solution contained the following composition: 22 mM NaCl, 110 mM CholineCl, 5 mM D-glucose, 10 mM HEPES, 0.8 mM MgCl_2_, and 1.8 mM CaCl_2_, pH = 7.4 (NaOH). The peptide was administered by continuous perfusion, and 100 µM CdCl_2_ was used to inhibit calcium currents. To acquire the current-voltage (I-V) relationships of sodium channels in DRG cells, test potentials ranged from -120 to +90 mV in 10 mV steps from a holding potential of -120 mV using EPC-10 (HEKA, Germany). 

For recording potassium currents, the intracellular solution contained the following composition: 120 mM KCl, 1 mM MgCl_2_, 5 mM EGTA, 14 mM phosphocreatine disodium salt, and 5 mM Na_2_-GTP, pH = 7.2 (KOH), and the extracellular solution contained the following composition: 1.8 mM CaCl_2_, 135 mM CholineCl, 10 mM D-glucose, 10 mM HEPES, 1 mM MgCl_2_, and 4.5 mM KCl, pH = 7.4 (KOH). To acquire the current-voltage (I-V) relationships of potassium channels in DRG cells, test potentials ranged from -80 to +80 mV in 10 mV steps from a holding potential of -80 mV using EPC-10. 

For recording calcium currents, the intracellular solution contained the following composition: 120 mM CsCl, 1 mM MgCl_2_, 10 mM HEPES, 4 mM Mg-ATP, 0.3 mM Na_2_-GTP, 10 mM EGTA, pH = 7.2 (CsOH). The extracellular solution contained the following composition: 140 mM TEA-Cl, 2 mM MgCl_2_, 5 mM D-glucose, 10 mM HEPES, and 10 mM CaCl_2_, pH = 7.4 (NaOH). To acquire the current-voltage (I-V) relationships of calcium channels in DRG cells, test potentials ranged from -60 to +40 mV in 10 mV steps.

### Whole-cell patch clamp for HEK293 and ND7/23 cells

HEK293 and ND7/23 cells, which were obtained from the Cell Bank of the Chinese Academy of Sciences (Shanghai, China) with STR Authentication, were cultured in DMEM with 10% fetal bovine serum. Plasmids of human Na_V_1.3 (hNa_V_1.3), human Na_V_1.4 (hNa_V_1.4), human Na_V_1.7 (hNa_V_1.7), human Ca_V_1.2 (hCa_V_1.2) and human Ca_V_2.2 (hCa_V_2.2) were separately transfected into HEK293 cells using Lipofectamine 3000 (Invitrogen, America), while plasmids of human hNav1.8 and hNavβ1 were cotransfected into ND7/23 cells.

For recording sodium channel currents, the intracellular solution contained the following composition: 60 mM CsF, 50 mM CsCl, 10 mM NaCl, and 5 mM HEPES, pH = 7.4 (CsOH). The extracellular solution contained the following composition: 140 mM NaCl, 3.5 mM KCl, 1 mM MgCl_2_•6H_2_O, 2 mM CaCl_2_•2H_2_O, 10 mM D-glucose, 10 mM HEPES, and 1.25 mM NaH_2_PO_4_•2H_2_O, pH = 7.4 (NaOH). The sodium currents were induced by depolarization of -10 mV from a holding potential of -120 mV.

For recording calcium currents in HEK293 cells, the intracellular solution contained the following composition: 120 mM CsCl, 1 mM MgCl_2_, 10 mM HEPES, 4 mM Mg-ATP, 0.3 mM Na_2_-GTP, 10 mM EGTA, pH = 7.2 (CsOH), and the extracellular solution contained the following composition: 140 mM TEA-Cl, 2 mM MgCl_2_, 5 mM D-glucose, 10 mM HEPES, 10 mM CaCl_2_, pH = 7.4 (NaOH). The calcium currents of Ca_V_1.2 and Ca_V_2.2 were induced by a 400 ms depolarization of 10 mV from a holding potential of -60 mV.

### MTT cytotoxicity assay

The conotoxin Tx6.7 was examined for cytotoxic activities against ND7/23 cell lines. Cytotoxicity assays were carried out in vitro using MTT staining according to the procedures [ 19]. The peptide concentrations used were 0, 0.01, 0.1, 1, 10, and 100 μM for each well, respectively. Three separate experiments were carried out, and six replicate wells were used to determine each point. After 48 h of incubation, the cells were stained with MTT and placed in a BIO-RAD model 680 microplate reader to determine the absorbance at 490 nm. 

### Analgesic activity bioassays

All animal procedures were carried out according to the approved protocol (GDY2002208) of the Institutional Animal Care and Use Committee at Guangdong Medical University. Female Kunming mice (weight 18-22 g) were purchased from the Guangzhou University of Chinese Medicine Experimental Animal Center (NO. SYXK(Yue)2018-0182). Ziconotide (ω-conotoxin MVIIA) was used as a positive control, while artificial cerebrospinal fluid (aCSF) was used as a negative control. The aCSF contained the following composition: 48 mM NaCl, 3 mM KCl, 1.4 mM CaCl_2_, 0.8 mM MgCl_2_, 1.5 mM Na_2_HPO_4_, 0.2 mM NaH_2_PO_4_, and 250 mM NaHEPES, pH = 7.35. Thirty Kunming mice were randomly divided into three groups. Each mouse was intrathecally injected with 20 pmol Tx6.7 or Ziconotide. The pain threshold was set as the time from when mice were placed on the 55 °C hot plate to when they licked their foot. The pain threshold was tested at 12 h before drug administration and 0.5 h, 1 h, 2 h, 3 h, and 4 h after drug administration.

$$\text{Pain threshold increment percentage (\%)}=\;\frac{\text{Pain threshold after administration}\;-\;\text{Pain threshold before administration}\;}{\text{Pain threshold before administration}}\;\times 100\%$$

## Results

### Sequence identification of the O1-conotoxin Tx6.7

**Figure 1.**
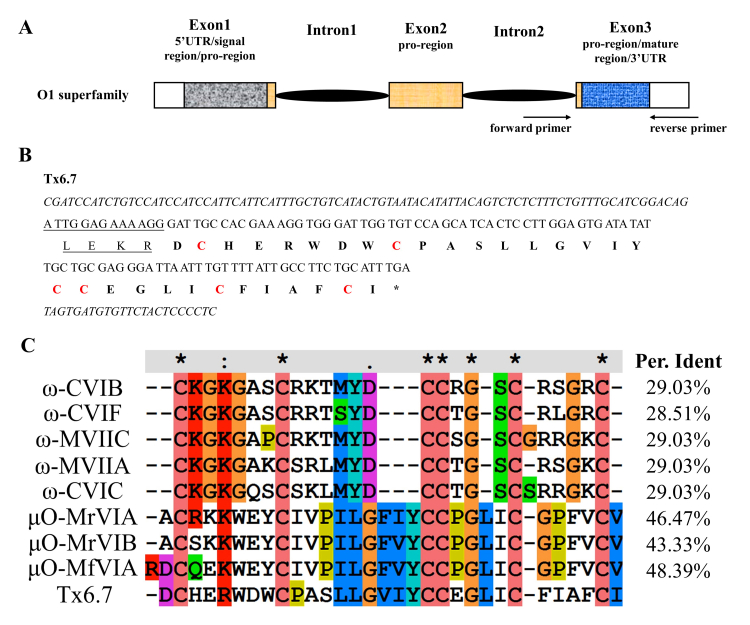
The sequence of Tx6.7. **(A)** The gene structure of the O1 superfamily. Different conotoxin gene segments are marked with different boxes. 5’ or 3’UTR: white box; signal region: gray box; pro-region: yellow box; mature region: blue box. **(B)** The genomic DNA sequence and predicted amino acid sequence of Tx6.7. The intron and 3’UTR sequences are in italics, the pro-region is underlined, the mature region is marked in bold and the six cysteines are marked in red. **(C)** Clustal alignment of nine O1 superfamily conotoxins. The alignment was performed by the software ClustalW.

The conotoxin Tx6.7 was cloned from the genomic DNA of Conus textile using primers designed based on the conserved 3’ end of the intron before the mature peptide region and the 3’ UTR elements in the O1 superfamily ( Fig. 1A). The cloned peptide sequence of Tx6.7 comprises 35 amino acid residues, including four amino acids at the end of the pro-region and 31 amino acids in the whole mature peptide ( Fig. 1B). According to the alignment of typical conotoxins in the O1 superfamily ( Fig. 1C), Tx6.7 has high sequence similarity with μO-MrVIA, MrVIB and MfVIA, and should have the same disulfide connectivity (I-IV, II-V and III-VI). 

### Peptide synthesis and identification of Tx6.7

**Figure 2.**
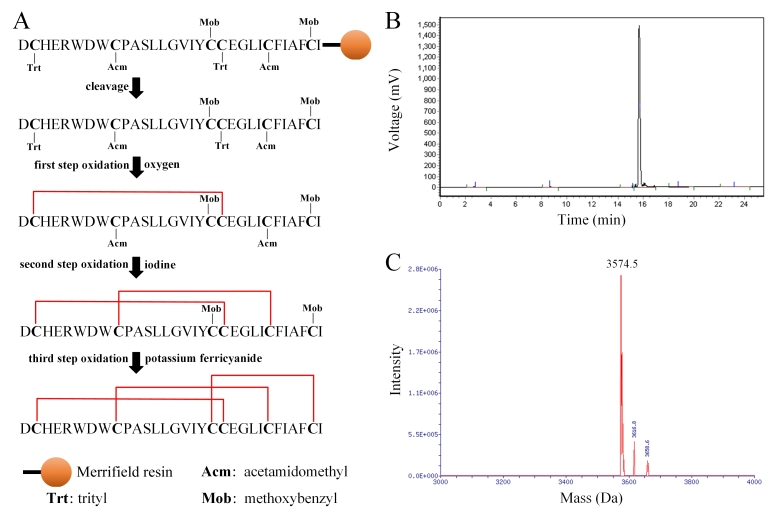
Synthesis and confirmation of Tx6.7. **(A)** Schematic representation of the disulfide bond synthesis of Tx6.7. **(B)** Purification of Tx6.7 by reverse-phase HPLC. **(C)** Mass spectra of Tx6.7.

The O1-conotoxin Tx6.7 was synthesized by solid-phase chemical synthesis according to its sequence (DCHERWDWCPASLLGVIYCCEGLICFIAFCI). Triphenylmethyl (Trt), acetamidomethyl (Acm), and methoxybenzyl (Mob) were used to protect the three pairs of cysteines separately, and oxidation of oxygen, iodine or potassium ferricyanide were used to form the three disulfide bonds ( Fig. 2A). The oxidized crude peptide was purified by reverse-phase HPLC ( Fig. 2B), and the molecular weight was determined by mass spectrometry ( Fig. 2C). Mass of the final peptide was 3574.5 Da, which was identical with the expected molecular weight, suggesting that conotoxin peptide Tx6.7 with three pairs of disulfide bonds was synthesized successfully. 

### Effects of Tx6.7 on sodium, potassium and calcium currents in DRG neurons

**Figure 3.**
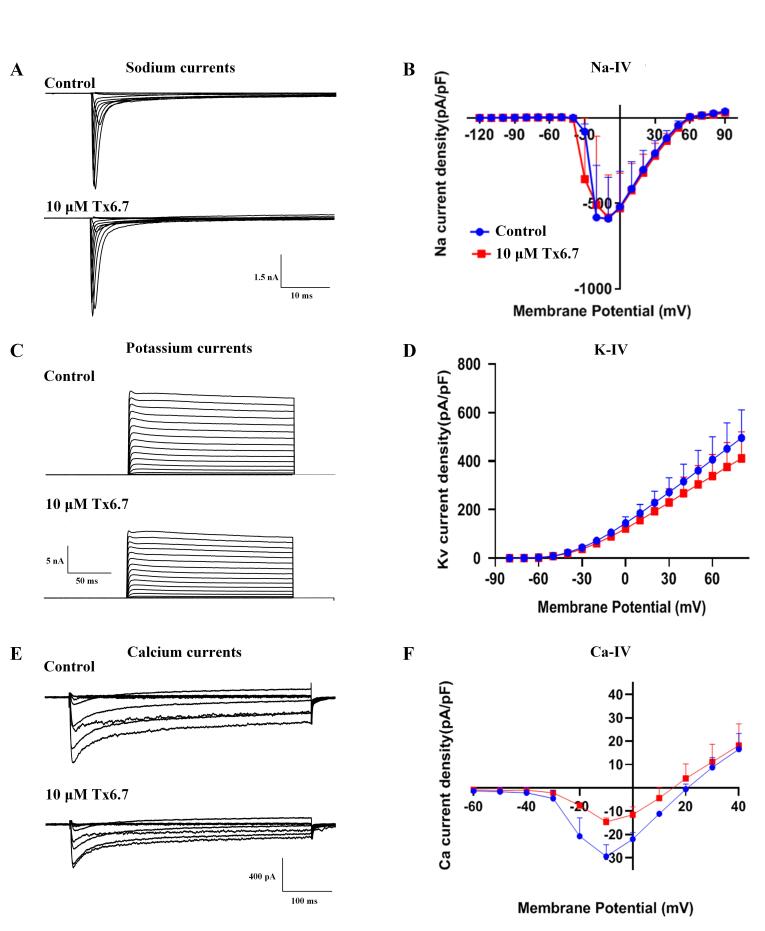
Effects of Tx6.7 on rat sodium, potassium and calcium currents. **(A)** Effects of 10 μM Tx6.7 on rat DRG sodium currents. **(B)** Effects of 10 μM Tx6.7 on the current-voltage (I-V) relationships of rat DRG sodium currents (n = 3). **(C)** Effects of 10 μM Tx6.7 on rat DRG potassium currents. **(D)** Effects of 10 μM Tx6.7 on the current-voltage (I-V) relationships of rat DRG potassium currents (n = 3). **(E)** Effects of 10 μM Tx6.7 on rat DRG calcium currents. **(F)** Effects of 10 μM Tx6.7 on the current-voltage (I-V) relationships of rat DRG calcium currents (n = 3). All error bars in the figures represent the standard error of the mean.

The electrophysiological effects of Tx6.7 on sodium, potassium and calcium channel currents were measured by patch clamp in acutely isolated rat DRG neurons. Tx6.7 (10 μM) had no inhibitory effects on the sodium currents in rat DRG neurons ( Fig. 3A) and did not induce a shift in the current-voltage relationship ( Fig. 3B, n = 3). Tx6.7 (10 μM) inhibited the potassium currents in rat DRG neurons ( Fig. 3C), and the peak currents were inhibited by 22.33 ± 7.81% ( Fig. 3D, n = 3, *p < 0.01*). Tx6.7 (10 μM) inhibited the calcium currents in rat DRG neurons ( Fig. 3E), and the peak currents were inhibited by 59.29 ± 2.34% ( Fig. 3F, n = 3, *p < 0.01*). Tx6.7 did not induce a shift in the current-voltage relationship of calcium currents ( Fig. 3F). 

### Effects of Tx6.7 on sodium and calcium channel subtypes

**Figure 4.**
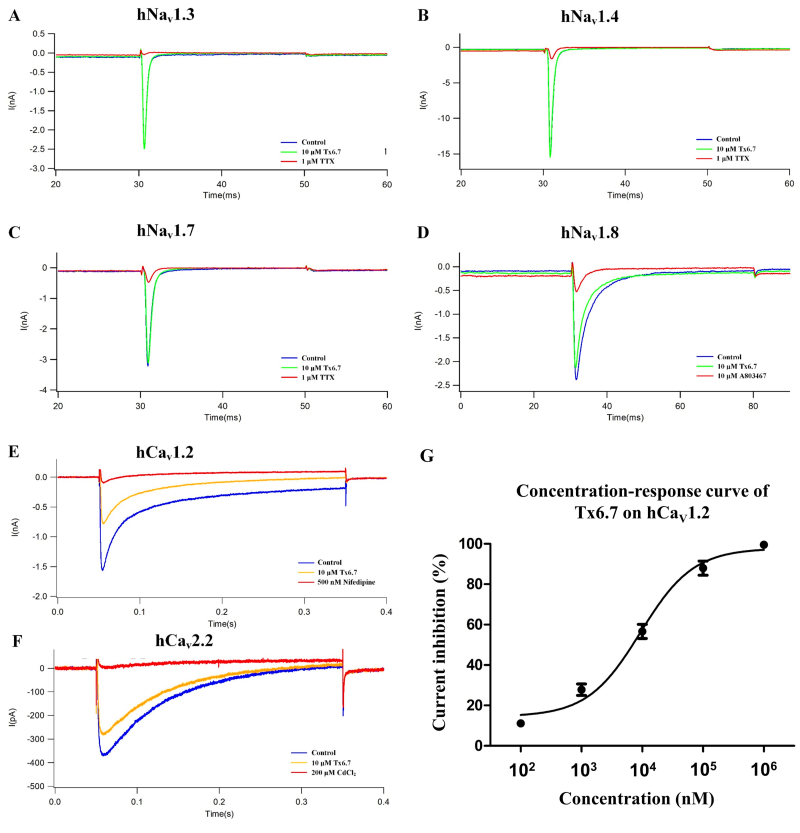
Effects of Tx6.7 on sodium and calcium channel subtypes. Effects of 10 μM Tx6.7 on hNa_V_1.3 **(A)**, hNa_V_1.4 **(B)**, hNa_V_1.7 **(C)** in HEK293 cells and hNa_V_1.8 **(D)** in ND7/23 cells. Circa 1 μM TTX was used as a positive control in the hNa_V_1.3, hNa_V_1.4 and hNa_V_1.7 tests. 10 μM A803467 was used as a positive control in the hNa_V_1.8 test. Effects of 10 μM Tx6.7 on hCa_V_1.2 **(E)** and hCa_V_2.2 **(F)** in HEK293 cells. 500 nM Nifedipine and 100 μM CdCl_2_ were used as positive controls in the hCa_V_1.2 and hCa_V_2.2 tests, respectively. ( **G**) The concentration-response curve of Tx6.7 on hCa_V_1.2 currents.

Plasmids of hNa_V_1.3, hNa_V_1.4, hNa_V_1.7, hCa_V_1.2 and hCa_V_2.2 were transfected into HEK293 cells respectively, while plasmids of human hNav1.8 and hNavβ1 were cotransfected into ND7/23 cells. Tx6.7 was then tested on these ion channel subtypes. For the sodium channel subtypes, Tx6.7 had no obvious effects on the hNa_V_1.3 ( Fig. 4A), hNa_V_1.4 ( Fig. 4B) and hNa_V_1.7 ( Fig. 4C) currents. About 10 μM Tx6.7 inhibited 7.30 ± 3.38% of the hNa_V_1.8 currents ( Fig. 4D, n = 3, *p < 0.01*). For the calcium channel subtypes, 10 μM Tx6.7 inhibited 56.61 ± 3.20% of the hCa_V_1.2 currents ( Fig. 4E, n = 3, *p < 0.01* ) and 24.67 ± 0.91% of the hCa_V_2.2 currents ( Fig. 4F, n = 3, *p < 0.01*). The IC_50_ value of Tx6.7 on hCa_V_1.2 currents was 9.63 ± 1.12 μM ( Fig. 4G).

### The cytotoxicity of Tx6.7

For the cytotoxicity experiments, the cell viability of ND7/23 cells incubated with different concentrations of Tx6.7 was measured by MTT ( Table 1). The cell viability values were more than 94% at all detected concentrations (0.01, 0.1, 1, 10, and 100 μM, p > 0.05), indicating that Tx6.7 had no significant cytotoxicity against ND7/23 cells up to 100 μM. 

**Table 1.** Cytotoxicity of Tx6.7 on ND7/23 cells. p values were calculated by the Student’s t-test.

Dose (μM)	0	0.01	0.1	1	10	100
OD490	0.742±0.074	0.756±0.062	0.779±0.094	0.736±0.078	0.732±0.063	0.703±0.061
Cell viability (%)	100	101.95	105.01	99.18	98.60	94.80

### The analgesic activity of Tx6.7

**Figure 5.**
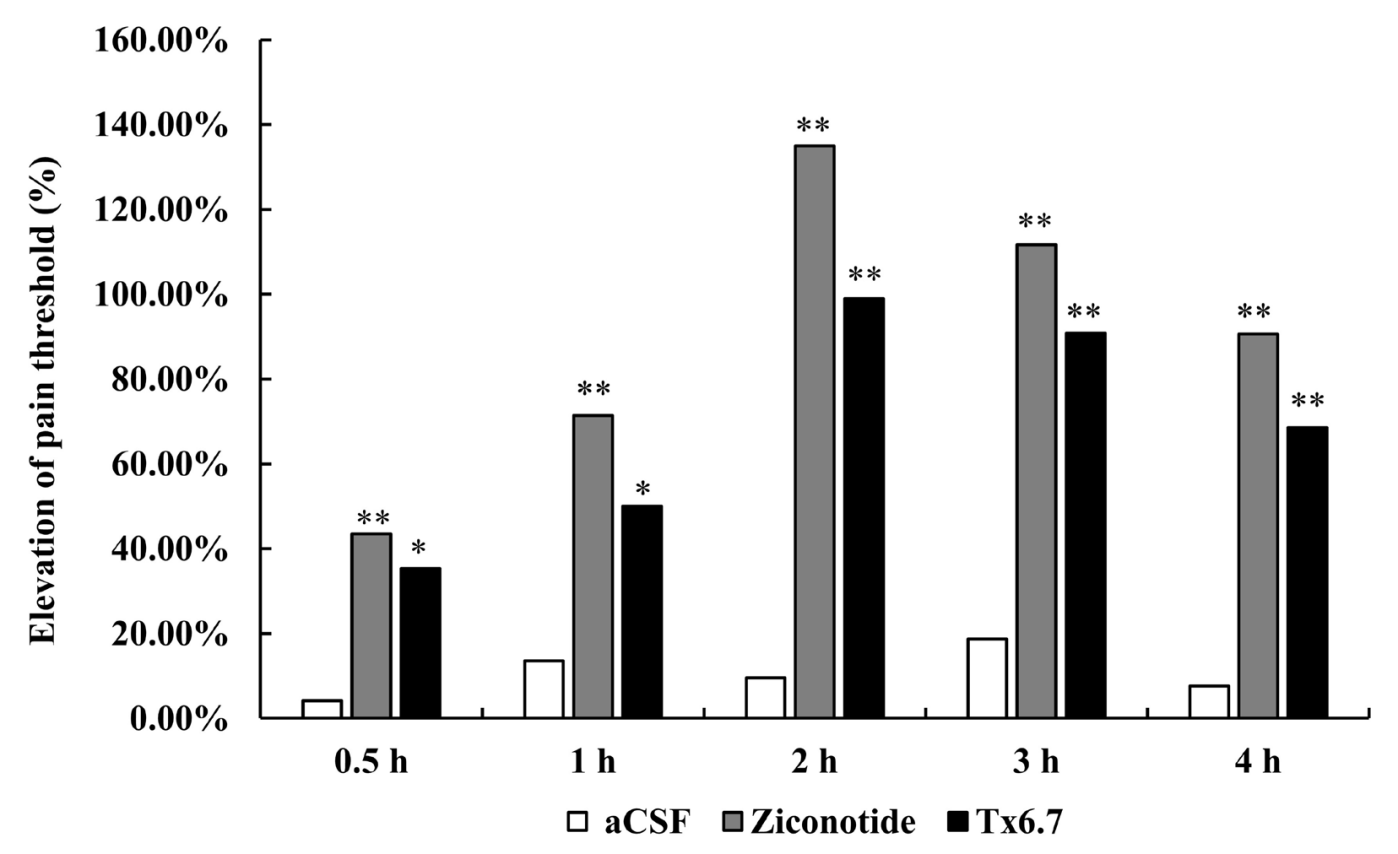
Analgesic effects of Tx6.7 tested by the mouse hot plate assay. The relationship between test time and the increased percentage of pain threshold (%) was shown. Values marked with asterisks are significantly different from the aCSF group. *p < 0.01, **p < 0.001.

The analgesic activity of Tx6.7 was evaluated by the mouse hot plate assay, which was tested at 0.5 h, 1 h, 2 h, 3 h, and 4 h after intrathecal injection ( Fig. 5). Ziconotide (ω-conotoxin MVIIA) was used as the positive control. The pain threshold was increased by 20 pmol Tx6.7 from 0.5 h to 4 h. The analgesic effects of Tx6.7 reached a maximum at 2 h with the pain threshold increasing by 98.91%. However, at each time point, Ziconotide showed a better analgesic effect than Tx6.7.

## Discussion

The traditional methods to discover a new conotoxin are peptide purification directly from the crude venom of Conus or gene amplification from the venom duct cDNA library. In this study, we successfully obtained a novel conotoxin using a new approach of genomic DNA cloning. The gene structures of nine conotoxin superfamilies were reported in previous work, and most of them have two introns and three exons [ 14]. According to the conserved sequences of the beginning and ending portions of the introns, we designed primers and cloned a new O1 superfamily conotoxin, Tx6.7, from the genomic DNA of Conus textile. Our results suggested that direct cloning of conotoxin sequences from the genomic DNA of cone snails would be an effective approach to obtain novel conotoxins. The drawback of this approach is that only exon3 (corresponding to the mature region) was obtained from this genomic PCR experiment ( Fig. 1). To obtain the signal and pro-regions, another two pairs of primers should be designed to clone the corresponding exon1 and exon2 sequences from two additional rounds of PCR experiments.

As one of the O1 superfamily conotoxins, Tx6.7 was compared with eight other ω/μO-conotoxins in the O1 superfamily ( Fig. 1C) [ 9, 18]. From the alignment result, Tx6.7 has high sequence similarity with these μO family conotoxins (MrVIA, MrVIB and MfVIA) which inhibit sodium currents, but has low sequence similarity with ω family conotoxins (CVIB, CVIB, MVIIC, MVIIA and CVIC) which inhibiting calcium currents. However, patch clamp experiments on DRG, HEK293 and ND7/23 cells showed that Tx6.7 had a low inhibitory effect on hNa_V_1.8 currents but significantly inhibited DRG calcium currents, hCa_V_1.2 and hCa_V_2.2 currents. The low sodium channel activity may be due to the lack of some key residues (such as the three prolines in MrVIA, MrVIB and MfVIA) relating to the inhibition of sodium currents in the amino acid sequence of Tx6.7. Point mutation of amino acid residues may improve the inhibitory effect of Tx6.7 on Na_V_1.8. 

Na_V_1.8 is selectively expressed in sensory neurons and plays an important role in neuropathic pain [ 20, 21]. Ca_V_2.2 is involved in the neurotransmitter release of nociceptive pathways from afferent terminals [ 22]. The inhibitory effects on these two channels suggested that Tx6.7 may have a certain analgesic effect. Ca_V_3.2 channels are the major isoform expressed in nonpeptidergic and peptidergic nociceptive neurons [ 23]. The patch clamp results showed that Tx6.7 inhibited 24.67 ± 0.91% of the Ca_V_2.2 currents. The observed analgesic activity in the hot plate test may be a result of Ca_V_3.2 inhibition, which would be verified in future work. Ca_V_1.2 channels are the main subtypes in ventricular cardiac muscle and are also present in the nervous system, secretory tissues and smooth muscle cells [ 24]. Inhibition of Ca_V_1.2 channels by specific blockers is a recognized pharmacodynamic approach for treating cardiac ischemia and hypertension [ 25], and Tx6.7 could be used as a candidate for this treatment. However, the action concentrations of Tx6.7 on Na_V_1.8, Ca_V_2.2 and Ca_V_1.2 are at the micromolar level, so the activity of Tx6.7 should be further improved by alanine scanning or other modifications.

## Conclusion

This study provided an unconventional approach to obtain novel conotoxin sequences from the genomic DNA of cone snails. The conotoxin Tx6.7 cloned from Conus textile could significantly inhibit rat DRG calcium currents. In addition, Tx6.7 inhibited 56.61 ± 3.20% of the hCa_V_1.2 currents and 24.67 ± 0.91% of the hCa_V_2.2 currents, indicating that it could be used as a probe tool for ion channel research or a therapeutic candidate for novel drug development.
